# An Efficient Total Synthesis of a Potent Anti-Inflammatory Agent, Benzocamphorin F, and Its Anti-Inflammatory Activity

**DOI:** 10.3390/ijms130810432

**Published:** 2012-08-21

**Authors:** Yu-Ren Liao, Ping-Chung Kuo, Jun-Weil Liang, Yuh-Chiang Shen, Tian-Shung Wu

**Affiliations:** 1 Department of Chemistry, National Cheng Kung University, Tainan 701, Taiwan; E-Mails: l3892101@mail.ncku.edu.tw (Y.-R.L.); l36994279@mail.ncku.edu.tw (J.-W.L.); 2 Department of Biotechnology, National Formosa University, Yunlin 632, Taiwan; E-Mail: pcckuoo@sunws.nfu.edu.tw; 3 National Research Institute of Chinese Medicine, Taipei 112, Taiwan; E-Mail: yuhcs@nricm.edu.tw; 4 Department of Pharmacy, China Medical University, Taichung 401, Taiwan; 5 Chinese Medicinal Research and Development Center, China Medical University Hospital, Taichung 401, Taiwan

**Keywords:** *Antrodia camphorata*, benzocamphorin F, anti-inflammatory

## Abstract

A naturally occurring enynyl-benzenoid, benzocamphorin F (**1**), from the edible fungus *Taiwanofungus camphoratus* (*Antrodia camphorata*) was characterized by comprehensive spectral analysis. It displays anti-inflammatory bioactivity and is valuable for further biological studies. The present study is the first total synthesis of benzocamphorin F and the developed strategy described is a more efficient procedure that allowe the large-scale production of benzocamphorin F for further research of the biological activity both *in vitro* and *in vivo*.

## 1. Introduction

**Figure 1:**
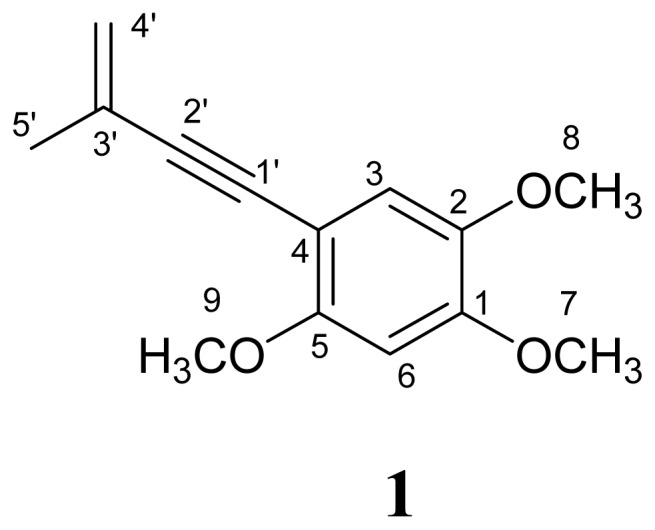
Structure of benzocamphorin F (**1**).

Inflammation is related to morbidity and mortality of many diseases and is recognized as part of the complex biological response of vascular tissues to harmful stimuli. It is the host response to infection or injury, which involves the recruitment of leukocytes and the release of inflammatory mediators, including nitric oxide (NO). NO is the metabolic by-product of the conversion of l-arginine to l-citrulline by a class of enzymes termed NO synthases (NOS). Numerous cytokines can induce the transcription of inducible NO synthase (*i*NOS) in leukocytes, fibroblasts, and other cell types, accounting for enhanced levels of NO. Although NO is a microbicide and may have important roles in tissue adapting to inflammatory states, overproduction of NO may exacerbate tissue injury in both acute and chronic inflammatory conditions. In the experimental model of acute inflammation, inhibition of *i*NOS can have a dose-dependent protective effect, suggesting that NO promotes edema and vascular permeability. NO also has a detrimental effect in chronic models of arthritis, whereas protection is seen with *i*NOS inhibitors [1,2]. For example, glucocorticoids, which are often used in the treatment of inflammation, are able to inhibit the expression of *i*NOS. Therefore, the extracts of *Taiwanofungus camphoratus*, which are considered as a rich source of triterepnoids, were examined for their anti-inflammatory activity to explore the new lead drugs. *T. camphoratus* (synonyms: *Ganoderma camphoratum; Antrodia cinnamomea; Antrodia camphorata*) (Polyporaceae, Aphyllophorales) is a rare and precious medical fungus in Taiwan and is called a “national treasure of Taiwan” with the Chinese name of Zhan-Ku or Niu-Chang-Chih [3]. The microorganism is parasitic to the inner heartwood wall of old hollow trunks of *Cinnamomum kanehirai* Hay. (Lauraceae). In traditional Taiwanese folk medicine *T. camphoratus* has been used as an important health food for treating food, alcohol, and drug intoxication, diarrhea, abdominal pain, hypertension, itching, and liver cancer [4,5]. Previous studies have revealed that Niu-Chang-Chih exerts various biological activities, such as hepatoprotective, antihepatitis B virus, anticancer, antioxidant, and anti-inflammatory activities [6,7]. The chemical constituents of this fungus can be divided into the three classes of polysaccharides, triterpenoids, and enynyl-benzenoids [8,9]. Our ongoing study on the chemical composition of the ethanol extract of the fruiting body of *T. camphoratus* has led to the isolation of one new enynyl-benzenoid, benzocamphorin F (**1**) (Figure 1). In the present study, we wish to report the structural determination of benzocamphorin F (**1**), as well as an evaluation of its anti-inflammatory activity. In addition, we developed a more efficient synthetic protocol that allowed the large-scale production of benzocamphorin F for the further research of its biological activity.

## 2. Results and Discussion

### 2.1. Isolation and Structural Elucidation

The ether-soluble fraction of the crude extract acquired for this study was successively subjected to column chromatography to yield benzocamphorin F (**1**). The structure of compound **1** was elucidated by the methods of UV, IR, HR-ESI/MS, ESI-MS/MS and NMR.

**Figure 2:**
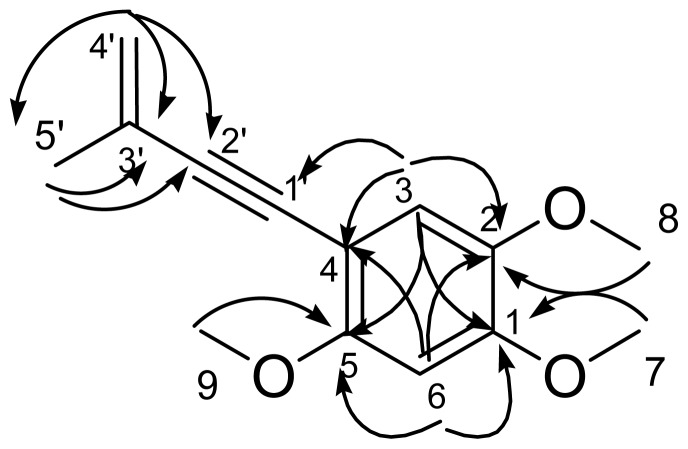
Heteronuclear multiple-bond correlation (HMBC) (→) correlations for benzocamphorin F (**1**).

Benzocamphorin F (**1**) was isolated as colorless powder and showed a [M + Na]^+^ ion peak at *m*/*z* 255.0997 in its HRESIMS, corresponding to the molecular formula C_14_H_16_O_3_Na. The UV spectrum of **1** displayed absorption maxima at 246, 258, 282 and 317 nm, and the IR spectrum exhibited strong absorption peaks for carbon-carbon triple bond (2183 cm^−1^), and carbon-carbon double bond (1605 cm^−1^), respectively. The ^1^H NMR (CDCl_3_) spectra of **1** showed signals assignable to a set of single aromatic protons at δ 6.91 (1H, s, H-3), 6.48 (1H, s, H-6), terminal methylene protons at δ 5.38 (1H, s, H-4′) and δ 5.26 (1H, s, H-4′), three methoxy singlets at δ 3.90 (3H, H-5), 3.88 (3H, H-1) and 3.84 (3H, H-2), and a methyl singlet at δ 2.02 (3H, s, H-3′), respectively. The ^13^C NMR and DEPT spectra combined with heteronuclear multiple-quantum correlation (HMQC) experiment indicated 14 signals including an olefinic carbon resonances at δ 121.2, three methoxy groups at δ 56.0, 56.4 and 56.9, two aromatic methines at δ 97.4 and 115.9, a methyl group at δ 23.6, seven quaternary carbons at δ 155.3, 150.3, 142.9, 127.1, 103.4, 93.5 and 84.7. The heteronuclear multiple-bond correlations (HMBC) (Figure 2) from OCH_3_-7(δ 3.88) to C-1(δ 155.3), from OCH_3_-8(δ 3.84) to C-2 (δ 142.9), from H-3(δ 6.91) to C-1(δ 155.3)/C-2(δ 142.9)/C-4(δ 103.4)/C-5(δ 150.3), from OCH_3_-9(δ 3.90) to C-5 (δ 150.3), from H-6 (δ 6.48) to C-1 (δ 155.3)/C-2 (δ 142.9)/C-4 (δ 103.4)/C-5 (δ 150.3), from CH_3_-5′ (δ 2.00) to C-2′ (δ 93.5)/C-3′ (δ 127.0)/C-4′ (δ 121.2), from H-4′ (δ 5.38, 5.26) to C-2′ (δ 93.5)/C-3′ (δ 127.0)/CH_3_-3′ (δ 23.6) constructed the substituted pattern of this enynyl-benzenoid. On the basis of these spectral data (Table 1), the chemical structure of **1** was identified as shown in Figure 1. It is the first report of this compound from the natural sources and it was given the trivial name, benzocamphorin F, proposed following a previous convention [9].

### 2.2. Chemistry

**Figure 3:**
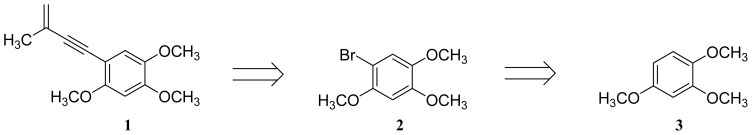
Retrosynthetic analysis of benzocamphorin F (**1**).

**Figure 4:**
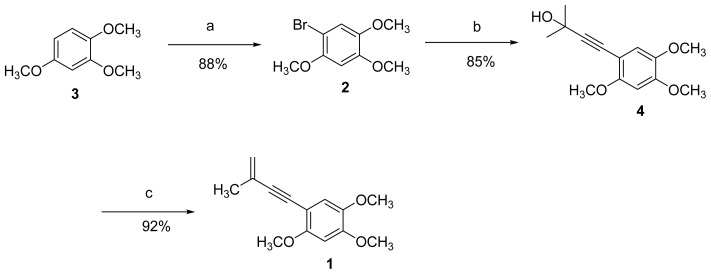
Synthesis of benzocamphorin F (**1**). **Reagents and conditions: a**) NBS, acetonitrile, room temp; **b**) 2-methyl-3-butyn-2-ol, Pd(PPh_3_)_4_, CuI, DMF; **c**) methanesulfonyl chloride, toluene, microwave.

In the previous literature, Wu *et al.* reported a total synthesis of antrocamphin A with six steps and an overall yield of 3.7% [10]. However, the low yield and high cost of the reagents for this method reduce the application efficiency. Herein we wish to explore a more efficient and economic method to prepare the analogs possessing the same skeleton as that of antrocamphin A. The retro-synthetic analysis of benzocamphorin F (**1**) was displayed in Figure 3 and thus we initiated the preparation of **1** from 1,2,4-trimethoxybenzene (**3**). The *N*-Bromosuccinimide (NBS)-assisted bromination of **3** resulted in the 1-bromo-2,4,5-trimethoxybenzene **2** as shown in Figure 4 [11]. Compound **2** was coupled with 2-methyl-3-butyn-2-ol by Sonogashira reaction [12], which successively led to compound **4**. Finally the dehydration of compound **4** in toluene by methanesulfonyl chloride to produce benzocamphorin F (**1**) in good yield (92%) in Figure 4 [13]. The chemical structure of synthetic compound **1** was elucidated by 1D and 2D-NMR and mass spectrometry and compared with those of the purified natural compound. The present synthetic protocol of benzocamphorin F (**1**) provides a more efficient synthetic pathway with a satisfactory overall yield (three steps, 68.9%). In addition, the reagents used in this synthesis were of comparatively low cost, thus it would be more suitable for the large-scale production of benzocamphorin F and the analogs with similar skeleton.

### 2.3. Anti-Inflammatory Activity

The quantity of purified benzocamphorin F (**1**) from natural sources was too small to be examined for bioactivity. Thus only the inhibitory effect of synthetic benzocamphorin F (**1**) on LPS-induced NO production in murine microglial cells (BV2) was investigated. Nitrite accumulated in the culture medium was estimated by the Griess reaction as an index for NO release from the cells [12]. When BV2 were treated with different concentrations of **1** together with LPS (0.5 μg/mL) for 24 h, a significant concentration-dependent inhibition of nitrite production was detected. The IC_50_ value for inhibition of nitrite production of **1** was 8.6 ± 2.7 μM. It was more potent than L-NAME (IC_50_: 12.0 ± 0.6 μM), a non-specific NOS inhibitor. To further understand whether **1** also exerted anti-oxidative properties, its effect on NOX activity was examined. Our data suggest that compound **1** (IC_50_: 74.4 ± 9.5 μM) was an ordinary inhibitor of NOX, as compared to the specific NOX inhibitor, DPI (IC_50_: 0.96 ± 0.06 μM) (Table 2), indicating that NOX might not be the direct target. The free radical-scavenging capacity of compound **1** was also examined in a cell-free DPPH solution and it did not show considerable free radical-scavenging activity. Trolox, a vitamin E analogue included as a positive control, displayed a stronger free radical-scavenging effect than the examined compound (Table 2).

## 3. Experimental Section

### 3.1. General

Melting points were determined using the Yanagimoto MP-S3 micro melting point apparatus without correction. Optical rotations were measured using a Jasco DIP-370 digital polarimeter. UV spectra were obtained on a Hitachi UV-3210 spectrophotometer, and IR spectra were recorded on a Shimadzu FT-IR DR-8011 spectrophotometer. ^1^H NMR (400 MHz) and ^13^C NMR (75 MHz) spectra were recorded on Bruker AMX-400 spectrometers using CDCl_3_ as the solvents. Chemical shifts are shown in δ values (ppm) with tetramethylsilane as an internal standard. ESI and HR-ESI mass spectra were measured on a Bruker APEX II mass spectrometer. Microwave irradiation was carried out using the CEM LabMate microwave apparatus. Reversed-phase column chromatography was accomplished with Diaion HP-20 and Sephadex LH-20 columns. Silica gel column chromatography was carried out using Kieselgel 60 (70–230 and 230–400 mesh, Merck). Thin-layer chromatography (TLC) was executed on pre-coated Kieselgel 60 F_254_ plates (Merck), with compounds visualized by UV light or spraying with 10% (*v*/*v*) H_2_SO_4_ followed by charring at 110 °C for 10 min.

### 3.2. Extraction and Isolation

The fruiting body of *T. camphoratus* (1.0 kg) was extracted with Et_2_O (4 × 10 L) for three days. The Et_2_O extract was concentrated to afford a brown syrup (360 g) and then partitioned between H_2_O and Et_2_O. The ether layer was chromatographed on silica gel and eluted with MeOH in chloroform (0–100% of MeOH, gradient) to obtain eight fractions, (Fr. A–H) monitored by TLC. Fr. C was chromatographed on a column of silica gel, eluted successively with a gradient of petroleum *n*-hexane–EtOAc (3:1 to 1:2) as eluent to yield **1** (18.0 mg). Fr. H was chromatographed on a column of silica gel, eluted successively with a gradient of petroleum *i*-Pr_2_O–EtOAc (15:1) as eluent to yield **2** (24.0 mg).

### 3.3. Preparation of the Compound (**1**)

#### Synthesis of 1-bromo-2,3,5-trimethoxybenzene (2)

A mixture of **3** (2.0 g, 11.9 mmol), *N*-Bromosuccinimide (2.1 g, 11.9 mmol) in MeCN (30 mL) was stirred in an ice bath, and the reaction mixture was checked by TLC. The mixture was diluted with water (50 mL) and then extracted by EtOAc (100 mL × 3). The combined organic phases were washed with water and brine, and further dried over anhydrous MgSO_4_. The crude was purified by column chromatography over silica gel using *n*-hexane:EtOAc (30:1) to obtain **2** (2.6 g, 88%) as pale yellow powder. **2**: ^1^H NMR (400 MHz, CDCl_3_) δ:7.03 (1H, s, H-3), 6.56 (1H, s, H-6), 3.88 (3H, s, OCH_3_-5), 3.86 (3H, s, OCH_3_-1), 3.83 (3H, s, OCH_3_-2); ^13^C NMR (75 MHz, CDCl_3_) δ: 150.3, 149.1, 143.8, 116.4, 101.1, 98.9, 57.2, 56.6, 56.2; HR-ESI-MS *m*/*z* 268.9798 [M + Na] (calcd for C_9_H_9_BrO_3_, 245.9982).

#### Synthesis of 2-methyl-4-(2,4,5-trimethoxyphenyl)but-3-yn-2-ol (4)

A mixture of **2** (0.5 g, 2.0 mmol), 2-methyl-3-butyn-2-ol (0.26 g, 3.1 mmol), Pd(PPh_3_)_4_ (5% eq) and CuI (10% eq) in DMF (15 mL) was stirred at reflux and the reaction mixture was checked by TLC. The mixture was diluted with water (50 mL) and then extracted by EtOAc (100 mL × 3). The combined organic phases were washed with water and brine, and further dried over anhydrous MgSO_4_. The crude was purified by column chromatography over silica gel using *n*-hexane:EtOAc (3:1) to obtain **4** (0.43 g, 85%) as pale yellow oil. 4: ^1^H NMR (400 MHz, CDCl_3_) δ:6.87 (1H, s, H-3), 6.47 (1H, s, H-6), 3.89 (3H, s, OCH_3_-5), 3.85 (3H, s, OCH_3_-1), 3.83 (3H, s, OCH_3_-2), 1.65 (6H, s, CH_3_-3′); ^13^C NMR (75 MHz, CDCl_3_) δ: 150.3, 149.1, 143.8, 116.4, 101.1, 98.9, 98.3, 80.2, 65.8, 32.1, 57.2, 56.6, 56.2; HR-ESI-MS *m*/*z* 273.1198 [M + Na] (calcd for C_14_H_18_O_4_, 250.1205).

#### Synthesis of benzocamphorin F (1)

A mixture of **4** (0.5 g, 2.0 mmol) and methanesulfonyl chloride (0.35 g, 3 mmol) in toluene (10 mL) was stirred by microwave r at 100 °C, 100W for 8 min (2 min ramp time followed by 6 min at 100 °C) and the reaction mixture was checked by TLC. The mixture was diluted with water (50 mL) and then extracted by EtOAc (100 mL × 3). The combined organic phases were washed with water and brine, and dried over anhydrous MgSO_4_. The crude was purified by column chromatography over silica gel using *n*-hexane:EtOAc (15:1) to obtain **1** (0.43 g, 92%).

### 3.4. Microglial Cell Culture and Measurement of Nitric Oxide (NO)

A murine microglial cell line (BV2) was cultured in Dulbecco’s modified Eagle medium (DMEM; Gibco, USA) supplemented with 5% fetal bovine serum (Hyclone, Logan, UT, USA). The production of NO was determined by measuring the accumulation of nitrite in the culture medium 24 h after stimulation with LPS (0.5 μg/mL) by the Griess reagent as in our previous report [14].

### 3.5. Measurement of NADPH Oxidase Activity

NADPH oxidase activity was measured as described previously [14]. Test compounds were added to the wells of a bioluminescence plate and incubated with 50 μg of cell homogenate for 20 min at 37 °C in the dark. O_2_^−^ production was stimulated with 200 μM NADPH, and the chemiluminescence was monitored for 30 min, after which the AUC (area under the curve) was calculated to represent reactive oxygen species production (NADPH activity).

### 3.6. Measurement of DPPH Radical Scavenging Capacity

A DPPH radical scavenging capacity assay was performed as in our previous report [12]. The compound was diluted with MeOH into a range of concentrations (0.1–50 μM). DPPH (Sigma-Aldrich, USA) solution (200 μL, final concentration: 200 μM in MeOH) was added to 10 μL of each diluted sample in a 96-well microplate, and the resulting solution was allowed to react for 30 min in the dark at ambient temperature. The absorbance caused by the DPPH radical at 517 nm was determined by a microplate-spectrophotometer. The radical scavenging capacity is expressed as delta OD_517_ (ΔOD_517_), and values are the means of three replicates. An antioxidant, Trolox (OXIS, USA), was included as a reference.

## 4. Conclusions

In summary, a new benzenoid, benzocamphorin F (**1**), was isolated from the fruiting body of *Taiwanofungus camphorates* for the first time. The structure of **1** was fully elucidated by 2D-NMR analysis and also by an efficient and simple method for chemical synthesis. This novel method has several advantages over the other methods, such as high yield, fewer reaction steps, faster reaction rate, and easy work-up without producing any significant by-product. The bioactivity examination results indicated that compound **1** displayed potent NO-reducing activities in microglial cells and thus it had potential to be an anti-inflammatory drug for the treatment of NO-dependent neurodegenerative disorders.

## Figures and Tables

**Table 1:** The ^1^H and ^13^C NMR chemical shifts of compound **1** in CDCl_3_.

Positions	δH (ppm)	δC (ppm)	HMBC
1	/	155.3 (C)	
2	/	142.9 (C)	
3	6.91 (s, 1H)	116.0 (CH)	C-1, C-2, C-4, C-5, C-1′
4	/	103.4 (C)	
5	/	150.3 (C)	
6	6.48 (s, 1H)	97.4 (CH)	C-1, C-2, C-4, C-5
7	3.88 (s, 3H)	56.9 (CH_3_)	C-1
8	3.84 (s, 3H)	56.4 (CH_3_)	C-2
9	3.90 (s, 3H)	56.0 (CH_3_)	C-5
1′	/	93.5 (C)	
2′	/	84.7 (C)	
3′	/	127.0 (C)	
4′	5.26 (s, 1H) 5.38 (s, 1H)	121.2 (CH_2_)	C-2′, C-3′, C-5′
5′	2.00 (s, 3H)	23.6 (CH_3_)	C-2′, C-3′, C-4′

**Table 2:** Summary of the effects of benzocamphorin F on nitric oxide synthase (NOS), NADPH oxidase (NOX) activity in murine microglial cells and DPPH assay.

Compound	IC_50_ (μM)		

	NOS	NOX	DPPH
**1**	8.6 ± 2.7 *	74.4 ± 9.5	NA
l-NAME	12.0 ± 0.6	NA	NA
DPI	NA	0.96 ± 0.06	NA
Trolox	NA	NA	21.3 ± 1.6

NOX and NOS activity were measured by ROS and NO production, respectively, in the presence of 1–50 μM of drugs. DPI (diphenyleneiodonium, a NOX inhibitor) and L-NAME (a NOS inhibitor) were included as positive controls. Data were calculated as 50% inhibitory concentration (IC_50_) and expressed as means ± SEM from 5 to 6 experiments performed on different days using cells from different passages.

* *p* < 0.05 as compared with relative positive control, respectively.
